# Operative management of midshaft clavicle fractures demonstrates better long-term outcomes: A systematic review and meta-analysis of randomised controlled trials

**DOI:** 10.1371/journal.pone.0267861

**Published:** 2022-04-29

**Authors:** Michael Zhipeng Yan, Wing-sze Yuen, Sung-ching Yeung, Christie Wong Wing-yin, Sonia Choi-ying Wong, Walter Wang Si-qi, Elaine Tian, Shireen Rashed, Colin Shing Yat Yung, Christian Xinshuo Fang

**Affiliations:** Department of Orthopaedics and Traumatology, The University of Hong Kong, Queen Mary Hospital, Hong Kong Special Administrative Region, Hong Kong, China; Assiut University Faculty of Medicine, EGYPT

## Abstract

**Introduction:**

Midshaft clavicular fractures are common amongst young adults. Conservative or surgical treatment for definitive fracture management has been widely debate, both with their pros and cons. Previous meta-analyses compared the clinical outcomes between conservative and surgical treatment options of midshaft clavicular fractures but failed to elucidate any difference in functional improvement. We postulate that functional improvement after fracture union plateaus and the clinical outcome after treatment varies at different time points. This meta-analysis will focus on the synthesis comparison of outcomes at early, short-term results (3 months), intermediate-term (6 to 12 months) and long-term (>24 months) clinical outcomes.

**Methods:**

A systematic search was done on databases (Pubmed, Embase, Medline, Cochrane) in June 2021. Search keywords were: midshaft clavicular fractures and clinical trials. Clinical trials fulfilling the inclusion criteria were selected for comparison and the clinical outcomes of midshaft clavicular fractures using surgical and non-surgical interventions in terms of improvement in the Disabilities of the Arm, Shoulder and Hand (DASH) score, Constant-Murley Score (CMS), time to union and risk ratio of treatment related complications were analysed in correlation with post-treatment timeframe.

**Results:**

Of the 3094 patients of mean age 36.7 years in the 31 selected studies, surgical intervention was associated with improved DASH score (standard-mean difference SMD -0.22, 95% CI -0.36 to -0.07, p = 0.003; mean difference MD -1.72, 95% CI -2.93 to -0.51, p = 0.005), CMS (SMD 0.44, 95% CI 0.17–0.72, p = 0.001; MD 3.64, 95% CI 1.09 to 6.19, p = 0.005), time to union (non-adjusted SMD -2.83, 95% CI -4.59 to -1.07, p = 0.002; adjusted SMD -0.69, 95% CI -0.97 to -0.41, p<0.001) and risk ratio of bone-related complications including bone non-union, malunion and implant failure (0.21, 95% CI 0.1 to 0.42; p<0.001). Subgroup analysis based on time period after treatment showed that surgical intervention was far superior in terms of improved DASH score at the intermediate-term results (6–12 months later, SMD -0.16, 95% CI -0.30 to -0.02, p = 0.02; and long term results (>24 months SMD -4.24, 95% CI -7.03 to -1.45, p = 0.003) and CMS (>24 months, SMD 1.03, 95% CI 0.39 to 1.68, p = 0.002; MD 5.77, 95% CI 1.63 to 9.91, p = 0.006). Surgical outcome is independent of fixation with plates or intra-medullary nails.

**Conclusion:**

Surgical intervention was associated with better clinical outcomes compared with non-surgical approach for midshaft clavicular fractures in terms of improvement in functional scores DASH, CMS, time to union and fracture related complications, although not to the minimal clinically significant difference. Benefits in the long-term functional improvements are more pronounced.

## Introduction

Midshaft clavicular fracture occupies 2.6–4% of all adult fractures [1, 2]. It is a common injury among young adults with over one-third of all clavicular fractures occurring in adolescent male, and one-fifth in adolescent female [3]. The middle third of the clavicle is especially vulnerable to traumatic injury since it is the thinnest part of the clavicle without reinforcement protection by muscle and ligamentous attachments. Midshaft clavicular fractures can be classified by the Neer Classification or AO classification for risk stratification and management [4, 5]. Essentially, these classifications broadly classify them into non-displaced fractures (AO/OTA classification type A) which can be managed conservatively, while management for displaced fractures (AO/OTA classification type B) remains controversial. Absolute surgical indications often quoted include: 1) open fractures; 2) complete displacement of fracture ends with fragments greater than the width of the clavicular bone; 3) clavicle shortening more than 2cm or with an angulation of more than 30 degrees [6]. Relative surgical indications are: 1) significantly displaced fractures with shortening; 2) bone fragment pressure endangering soft tissue recovery; 3) lateral third clavicular fracture; 4) floating shoulder; 5) recurrent fractures; 6) non-union or malunion related complications.

Conservative treatment, such as the use of an arm sling immobilization followed by gentle range of motion exercises has been associated with significant non-union rates (range: 11–30%), poor cosmesis and decreased shoulder and strength and endurance [7–10]. However, the surgical decision is individualized based on the shoulder functional demand of patients since surgical management has its own disadvantages such as longer time for bone union, complications such as infection, neurovascular injury and the need for secondary procedures including implant removal [11, 12]. Clavicle implants have notoriously caused hardware impingement necessitating removal surgery in a high proportion of patients. Previous evidence does not support one management over the other. Early meta-analyses have summarised clinical trials comparing surgical interventions and conservative treatment but they show no functional differences between both interventions in short term; however, we postulate that surgical intervention leads to faster functional recovery compared to conservative means and better long-term prognosis [13–15]. Much of the patients attain fracture union and plateau in functional improvement with fair recovery in the long-term. Moreover, more detail on fracture fixation methods, approach and implants used may grossly affect the final outcome. This systematic review and meta-analysis aims at an updated comparison of the clinical outcomes between conservative treatment and surgical treatment of midshaft clavicular fracture at different time points (early, intermediate and late), and the subgroup analysis of management modalities within the surgical arm with the inclusion of most recent randomized control trials.

## Methods

A systematic search was performed on databases (Pubmed, Embase, Medline, Cochrane) in June 2021. Search keywords were: midshaft clavicular fracture and clinical trials. Clinical studies fulfilling the inclusion criteria were selected to evaluate the treatment efficacy of conservative treatment and surgical treatment in terms of change in functional scores; the Constant-Murley score (CMS), Disability Assessment of Shoulder and Hand (DASH) scores and visual analogous score of pain, time taken for radiological union and risk ratio of complication rates. The inclusion criteria for patients were: 1) closed midshaft clavicular fractures; 2) aged 18 or above; 3) with informed consent and be able to comply with follow-up period; 4) medically fit for surgery and anaesthesia. Exclusion criteria were: 1) fracture at proximal or distal third of the clavicle; 2) polytrauma or presentation delayed beyond 24 hours post-injury; 3) pathological fracture; 4) open fractures; 5) associated neurovascular injuries; 6) medical contraindications to surgery or high risk of anaesthesia.

Conservative treatment includes sling immobilization, figure-of-eight bandages, analgesics and rehabilitation exercises, while surgical management options include closed reduction with intramedullary fixation or open reduction internal fixation (ORIF) often utilizing different fixation methods. These include anatomical and non-anatomical locking clavicular plate fixation via superior or anterosuperior plating, dynamic compression plating with non-locking screws, titanium elastic intramedullary nailing and minimally invasive plate osteosynthesis. Subgroup analysis and qualitative systemic review was performed comparing different fixation methods.

### Data analysis

The primary aim of the review is to evaluate the efficacy of conservative treatment and surgical treatment for midshaft clavicular fractures. The primary outcome is the improvement functional scores (DASH and CMS) over time. DASH score is a function assessment to quantify the impact of the impairment on the level of arm, shoulder and hand–with a lower score signalling better functional outcome [16]. CMS is a 100-point scale that defines the level of pain and the ability to carry out normal daily activities of the patient with a higher score suggesting better functional return [17]. Previous studies have shown that the minimal clinically important difference for DASH and CMS were 10.83 points and 10.4 points, respectively [18, 19]. Secondary outcomes include time to fracture union confirmed by radiological investigations and risk ratios of complication rates including malunion, non-union, chronic neuropathy by the end of the study follow-up. Early outcome after treatment is defined as 3 months, intermediate outcome as between 6 and 12 months and late outcome is classified as 24 months after treatment.

**Fig 1:**
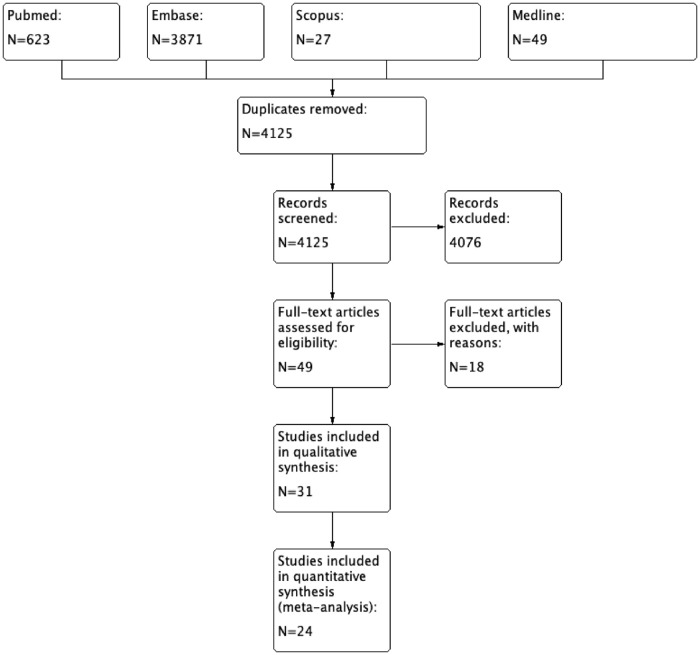
PRISMA study flow diagram.

The titles, abstracts and full articles were independently screened by two authors (ZPY and WSY). Following the PRISMA guidelines in PRISMA flow diagram, the study profile is shown in Fig 1. Duplicate articles were removed from analysis and articles were excluded if they were reviews, conference abstracts, research protocols or articles without primary therapeutic data.

Data extraction was performed with focus on study design, population demographics and therapeutic outcomes. Bias assessment was performed by Cochrane collaboration tool for randomised controlled trial (RCT). Bias or quality issues were minimized by cross-checking and inter-rater reliability test between authors, using IBM SPSS Statistics for Windows, version 28 (IBM Corp., Armonk, N.Y., USA). An inter-rater reliability test Cronbach’s Alpha of more than 0.7 as the acceptable inter-rater agreement [20]. Intraclass correlation coefficient was calculated with two-way mixed model of absolute agreement with a 95% confidence interval.

Review manager, version 5.3 and SPSS (IBM Corp., Armonk, N.Y., USA) were used in data analysis. Dichotomous data were pooled in random-effect model as a risk ratio with 95% confidence interval; while continuous data were pooled in random-effect model as a weighted average using generic inverse-variance method with 95% confidence interval. Heterogeneity was assessed with chi-square (χ^2^) test, with p-value smaller than 0.1 as statistically significant. Its’ extent was measured with *I*2-test. Egger’s test for funnel plot asymmetry was performed for assessment of publication bias for outcome measures with at least 10 studies.

## Results

As of June 2021, 4125 articles were retrieved from electronic databases (Pubmed, Embase, Scopus, Medline). After exclusion of duplicates and screening of titles and abstracts—articles were identified for full text review. A total of 49 articles were selected for eligibility assessment. 18 of them were excluded due to: (N = 10), protocol (N = 5) or non-English articles (N = 3). Eventually, 31 articles were selected for qualitative analysis. Amidst, 24 of them were included for meta-analysis review. Table 1 shows the summary of the pooled studies. Inter-rater variability is calculated with Cronbach’s alpha 0.826, and intraclass correlation coefficient is 0.815 (95% CI 0.681–0.898).

**Table 1:** Summary of the selected studies which fulfilled the inclusion criteria: 1) closed midshaft clavicular fractures; 2) aged 18 or above; 3) with informed consent and be able to comply with follow-up period; 4) medically fit for surgery and anaesthesia. Exclusion criteria were: 1) fracture at proximal or distal third of the clavicle; 2) polytrauma or presentation delayed beyond 24 hours post-injury; 3) pathological fracture; 4) open fractures; 5) associated neurovascular injuries; 6) medical contraindications to surgery or high risk of anaesthesia.

No.	References	Study design	Number of patients	Mean age & Sample size	Intervention arm; number of patients & mean follow-up duration	Control arm; number of patients & mean follow-up duration	Findings
1a) Selected studies comparing surgical intervention versus non-operative management.							
1	Bhardwaj 2018[21]	Single centre prospective RCT	69	32.1	Superior precontoured locking clavicle plate (LCP): 36 patients Follow up duration: 24 months	Non-operative arm pouch, 33 patients Follow up duration: 24 months	CSS were 89.42 ± 5.61 and 76.24 ± 3.43 in surgical intervention group and non-operative group, respectively. Time to union were 15.6 ± 0.8 and 22.8 ± 0.4 in surgical intervention group and non-operative group, respectively. Bone-related complications (malunion, non-union and implantation failure) occurred in 1 patient in surgical group, and 5 patients in control arm.
2	Canadian Trauma Society 2017[7]	Multicentre, prospective RCT	132	33.5	Superior clavicle LC-DCP, precontoured and reconstruction plates: 67 patients Follow up duration: 12 months	Non-operative sling, 65 patients Follow up duration: 12 months	Significantly improved DASH score and Constant Score in the operation group at all time points (p<0.01) Mean time to radiographic union was 16.4 weeks in the operative group, and 28.4 weeks in the non-operative group. (p = 0.001) 2 non-unions occurred in the operative group, compared with 7 in the non-operative group. (p = 0.001) Patients in the operative group were more likely to be satisfied with the shoulder cosmesis than those in the non-operative group (p = 0.001).
3	Altamimi 2008[22]	Multicentre, prospective RCT	132	Age unspecified	Superior clavicle DCP: 67 patients Follow up duration: 12 months	Non-operative sling, 65 patients Follow up duration: 12 months	CSS and DASHS were significantly improved in the operative group at all time points (p = 0.001 and p<0.01, respectively). Mean time to radiographic union was 16.4 weeks in the operative group, and 28.4 weeks in the non-operative group. (p = 0.001) Bone-related complications (malunion, non-union and implantation failure) occured in 2 patient in surgical group, and 7 patients in non-operative arm. One year after surgery, patients in operative group were more likely to be satisfied with their appearance of the shoulder (p = 0.001), compared with the non-operative group.
4	Woltz 2017[23]	Multicentre, prospective RCT	160 patients	45.5	Superior plate fixation with precontoured plate in superior, anterior and anterosuperior plating: 86 patients Follow up duration: 52 months	Non-operative sling, 74 patients Follow up duration: 55 months	Similar satisfaction score for surgical intervention and non-surgical sling. (p = 0.12) Bone-related complications (malunion, non-union and implantation failure) was significantly higher in non-operative arm: in 2.4% patient in surgical group, and 23.1% patients in non-operative arm. (p<0.0001)
5	Robinson 2013[11]	Multicentre, prospective RCT	200	32.4	Superior precontoured LCP: 95 patients Follow up duration: 12 months	Non-operative collar and cuff 105 patients Follow up duration: 12 months	Better CSS in Surgical group (92.0, 95% CI 90.0 to 94.0) than non-operative group (87.8 95% CI 85.2 to 90.3) followed up 1 year after surgery. Better DASHS in Surgical group (3.4, 95% CI 1.9 to 4.9) than non-operative group (6.1 95% CI 4.1 to 8.1) followed up 1 year after surgery. Bone-related complications (malunion, non-union and implantation failure) occurred in 1 patient in surgical group, and 16 patients in non-operative arm.
6	Ahrens 2017[24]	Multicentre, prospective RCT	301	36.2	Precontoured LCP: 154 patients Follow up duration: 9 months	Non-operative sling, 147 patients Follow up duration: 9 months	No difference in radiographic non-union or malunion at 3 months between the operative (28%) and non-operative group (27%) Significantly fewer radiographic non-union patients at 9 months in operative group (0.8%) compared with non-operative group (11%). DASHS and CSS were significantly better in the operative group, compared with non-operative group.
7	Cole 2014[25]	Multicentre, prospective RCT	200	32.0	Superior clavicle LCP: 95 patients Follow up duration: 12 months	Non-operative sling, 105 patients Follow up duration: 12 months	Patients in the operative group had significantly better CSS (p = 0.01) and DASHS (p = 0.02) than those in the non-operative group at 3 months. Patients in the operative group had significantly better CSS (92.0 vs 87.8; p = 0.01) and DASHS (3.4 vs 6.1; p = 0.04) than those in the non-operative group at 12 months. Significantly lower non-union rate in operative group (1.2%), compared with non-operative group (17%). Relative risk reduction of non-union was 93% (95% CI 50–99) with number needed to treat was 6 (95% CI 4–12).
8	Tamouki 2017 [26]	Multicentre, prospective RCT	117	32.5	Anterior construction plate: 59 patients Follow up duration: 12 months	Non-operative figure-of-eight harness 58 patients Follow up duration: 12 months	No difference between the 2 groups in DASHS at any time point (p = 0.398, 0.403 and 0.877 at 6 weeks, 6 months and 1 year, respectively). Significantly lower non-union rate in surgical group (0%), compared with non-operative group (14.9%).
9	Schemitsch 2011[27]	Multicentre, prospective RCT	132	33.5	Superior clavicle plate, small fragment LCP: 67 patients Follow up duration: 26 months	Non-operative sling 65 patients Follow up duration: 26 months	No difference in 1 vs 2 years after follow up in both operative group (p = 0.63) and non-operative (p = 0.59) after follow up. No difference in CSS in 1 vs 2 year after follow up in both operative group (p = 0.34) and non-operative group (p = 0.73) after follow up.
10	Judd 2009[28]	Multicentre, prospective RCT	57	Age not specified	Surgical fixation with Haige pin: 29 patients Follow up duration: 12 months	Non-operative sling 28 patients Follow up duration: 12 months	One patient in each group developed non-union Better short-term shoulder function score in operative fixation group, but similar shoulder function was observed in 6 months and 1 year afterwards.
11	Ban 2021[29]	Multicentre, prospective RCT	120	37.5	Superior clavicle LCP: 60 patients Follow up duration: 12 months	Non-operative sling 60 patients Follow up duration: 12 months	Significant DASH score found in favour of operative group at 6 weeks. (p≪0.001) Good DASH and CMS in both operative and non-operative groups 12 months later. Significantly higher rate of non-union in non-operative group (p = 0.014) with a relative risk of 9.47 (95% CI, 1.26–71.53), compared with operative group
12	Dugar 2013[30]	Single centre prospective RCT	30	Age unspecified	Superior clavicle plate fixation: 15 patients Follow up duration: 12 months	Non-operative sling 15 patients Follow up duration: 12 months	DASH score significantly improved at all time-points in the operative group. Mean time of radiographic union was 27.46 weeks in the non-operative group, and 15.73 weeks in the operative group. (p<0.001) No non-union in both groups. No malunion in operative group, while 7 malunion cases in the non-operative group.
13	Virtanen 2012[31]	Multicentre, prospective RCT	60	36.7	Stainless steel reconstruction plate: 28 patients Follow up duration: 12 months	Non-operative sling 32 patients Follow up duration: 12 months	No difference in DASH score (p = 0.89) and the Constant Score (p = 0.75) 1 year after treatment. All fractures in the operative group healed, but 6 non-unions in the nonoperative group.
14	Chen 2011[32]	Multicentre, prospective RCT	60	38.7	Elastic Stable Intramedullary Nailing (ESIN) with titanium elastic nail fixation: 30 patients Follow up duration: 15 months	Non-operative sling 30 patients Follow up duration: 15 months	ESIN led to a shorter time to union for fractures. 15 months after the surgery, patients in the ESIN group were more satisfied with their shoulder appearance and functions, compared with non-operative group. Significantly lower DASH score and higher CMS in the operative group.
15	Smekal 2009[33]	Multicentre, prospective RCT	60	37.7	ESIN with Titanium elastic nail fixation: 30 patients Follow up duration: 24 months	Non-operative sling 30 patients Follow up duration: 24 months	Fracture union observed in all patients in operative group, while 3 non-unions occurred in non-operative group. Significantly lower DASH score and higher CMS in 6 months and 2 years after trauma in operative group. Patients in the operative group are more satisfied with the shoulder cosmetic and functional outcomes, compared with the non-operative group.
16	Abo EI Nor 2013[34]	Multicentre, prospective RCT	20	31.0	Intra-medullary fixation with partially threaded cancellous screws: 10 patients Follow up duration: 16 months	Non-operative sling 10 patients Follow up duration: 16 months	All fracture cases united within 7–9 weeks (mean 8.2)
17	Smekal 2011[35]	Multicentre, prospective RCT	112	37.4	ESIN with Titanium elastic nail fixation: 60 patients Follow up duration: 24 months	Non-operative sling 52 patients Follow up duration: 24 months	ESIN led to quicker fracture union and better restoration of clavicular length Functional outcome DASH score was better in the operative group. Delayed union or malunion accounted for the majority of complications in the non-operative group.
18	Ferran 2010[36]	prospective RCT	32	29.3	Locked intramedullary nail fixation with Rockwood pin method: 17 patients Follow up duration: 12.4 months	Superior plating with LC-DCP: 15 patients Follow up duration: 12.4 months	The mean Constant score was 92.1 for the Rockwood Pin group and 88.7 for the plating group. The mean Oxford score was 45.2 for the Rockwood Pin group and 44.7 in the plating group There was no significant difference in either Constant scores (P = .365) or Oxford scores (P = .773). There was 100% union in both groups
19	Van de Meijden 2015[37]	Multicentre, prospective RCT	120	39.0	ESIN with Titanium elastic nail fixation: 62 patients Follow up duration: 12 months	Anterosuperior locking plate fixation: 58 patients Follow up duration: 12 months	No significant differences in the Disabilities of the Arm, Shoulder and Hand (DASH) or Constant-Murley score(3.0 and 96.0 points for the plate group and 5.6 and 95.5 points for the nailing group) were noted between the two surgicalinterventions at six months postoperatively. Until six months after the surgery, the plate-fixation group experienced less disability than the nailing group as indicated by the area under the curve of the DASH scores for the fracture.
20	Andrade-Silva 2015[38]	prospective RCT	59	29.9	Superior non-locked reconstruction plate fixation: 26 patients Follow up duration: 12 months	ESIN with Titanium elastic nail fixation: 33 patients Follow up duration: 12 months	The mean six-month DASH score was 9.9 points in the plate group and 8.5 points in the nail group (p = 0.329). Similarly, there were no differences in the twelve-month DASH and Constant-Murley scores. Time to union was equivalent(p = 0.352) between the groups at 16.8 weeks for the plate group and 15.9 weeks for the nail group, whereas the residual shortening was 0.4 cm greater in the plate group (p = 0.032). The visual analog scale pain score and the satisfaction ratewere similar between the groups. Implant-related pain was more frequent in the nail group (p = 0.035). There were no differences in terms of major complication
21	Van de Meijden 2016[39]	prospective RCT	120	39.0	ESIN with Titanium elastic nail fixation: 62 patients Follow up duration: 12 months	Anterosuperior clavicle plate with non-locking screws: 58 patients Follow up duration: 12 months	The nonweighted STI after 6 weeks was significantly higher in the PF group. During further follow- up, the differences leveled out and became nonsignificant. When weighting the STI for severity, the indices decrease but are significantly in favor of the PF group at 6 weeks and 6 months after surgery. At 1 year postoperatively, differences are not significant.
22	Fuglesang 2017[40]	prospective RCT	123	35.5	ESIN with Titanium elastic nail fixation: 60 patients Follow up duration: 12 months	Superior clavicular plate: 63 patients Follow up duration: 12 months	Plate fixation provided a faster functional recovery during the first six months compared with ESIN, but there was no difference after one year. After 12 months, there was no difference in DASH score between the plate fixation and ESIN, with both approaching their DASH baseline values of 0.5. Individual differences between baseline data and the DASH score after one year showed no statistical difference (1.4, -4.2 to 12.1 for plate versus 2.0, -14.2 to 28.3 for ESIN; p = 0.5, independent samples t-test). The duration of surgery was shorter for ESIN (mean 53.4 minutes, 22 to 120) than for plate fixation (mean 69.7 minutes, 35 to 106, p < 0.001). The recovery after ESIN was slower with increasing fracture comminution and with open reduction (p < 0.05)
23	Narsaria 2014[41]	prospective RCT	66	39.5	ESIN with Titanium elastic nail fixation: 33 patients Follow up duration: 24 months	Precontoured clavicular dynamic compression plate: 32 patients Follow up duration: 24 months	Length of incision, operation time, blood loss and duration of hospital stay were significantly less for the EIN group. American Shoulder and Elbow Surgeons (ASES) and Constant Shoulder scores were significantly higher (p<0.05) in the plating group than the EIN group for the first 2 months but there was no significant difference found between the two groups regarding functional and radiological outcome at the 2-year follow-up. Significantly higher rates of refracture after implant removal (p = 0.045) in the plating group was observed. Infection and revision surgery rates were also higher in the plate group, but this difference was insignificant (p>0.05).
24	Assobhi 2011[42]	prospective RCT	38	31.5	Anterior plating with reconstruction plate: 19 patients Follow up duration: 12 months	ESIN with Titanium elastic nail fixation (retrograde insertion): 19 patients Follow up duration: 12 months	Similar results were found between the two groups regarding functional and radiological outcome after the 12th week (P>0.05). earlier union and functional recovery were obtained at the 6th week for the RTEN group (P<0.05). The rate of complications was significantly higher (15.8%) in the plate group compared with the RTEN group (0%; P>0.05). In the plate group, significantly higher values were obtained for the perioperative data (P<0.001).
25	Kim 2018([43]	prospective RCT	30	38.1	Minimally invasive plate osteosynthesis 15 patients Follow up duration: 13.33 months	Conventional Plate Osteosynthesis 15 patients Follow up duration: 13.73 months	The Constant score and the visual analog scale satisfaction score were higher in the minimally invasive plate osteosynthesis group than in the conventional plate osteosynthesis group there was no significant difference between the groups in these scores or in the time to bone union (all P>.05). Operative time (52.33±13.87 vs 110.33±25.39 minutes, P < .001) and scar length (64.95±3.19 vs 99.39±15.98 mm, P < .001) were significantly shorter in the minimally invasive plate osteosynthesis group than in the conventional plate osteosynthesis group
26	Jiang 2012[44]	prospective RCT	64	42.5	Minimally invasive plate osteosynthesis 32 patients Follow up duration: 15 months	Conventional open reduction 32 patients Follow up duration: 15 months	The mean time to union was 13 weeks in the open reduction group compared to 12 weeks in MIPPO group (P > 0.05). The MIPPO group had no significantly superior Constant shoulder scores or DASH scores at all time-points (P > 0.05) the complications in the open reduction group were dysesthesia in the area of the incision and directly below in 10 cases, hypertrophic scarring in five cases, painful shoulder in two cases and a limitation of shoulder motion in one case (P > 0.05). The complications in the MIPPO group were dysesthesia in two cases, no hypertrophic scarring, no painful shoulder, no limitation of shoulder motion were noted (P < 0.05).
1b) Intra-surgical comparison of selected studies comparing surgical plate versus nail							
27	Yuan 2020[45]	prospective RCT	163	Age unspecified	Minimally invasive plate osteosynthesis: 82 patients Follow up duration: 3 months	Intramedullary nail fixation with screws 81 patients Follow up duration: 3 months	At 3 months after surgery, Constant-Murley scores were significantly higher and DASH scores were significantly lower in the MIPO group than the IMN group. No significant difference was observed for both indexes at 6 months. The fracture nonunion rate was significantly lower in the MIPO group. No significant difference was found in other complications.
28	Hulsmans 2017[46]	prospective RCT	120	39.0	Plating: 58 patients Follow up duration: 39 months	Intramedullary nail fixation, unspecified: 62 patients Follow up duration: 39 months	there were no differences in QuickDASH score (plate, 1.8±3.6; intramedullary nail, 1.8±7.2; mean difference, -0.7; 95% CI, -2.2 to 2.04; p = 0.95). The proportion of patients having implant-related irritationwas not different (39 of 56 [70%] versus 41 of 62 [66%]; relative risk, 1.05; 95% CI, 0.82–1.35; p = 0.683). Intra-medullary fixation was associated with a higher likelihoodof implant removal (51 of 62 [82%] versus 28 of 56 [50%]; relative risk, 1.65; 95% CI, 1.24–2.19; p<0.001)
29	Calbiyik 2017[47]	prospective randomized two-arm study	75	40.5	Surgical plate fixation with LCP: 40 patients Follow up duration: 12 months	Intramedullary nail fixation with Sonoma Crx device: 35 patients Follow up duration: 12 months	Mean time of operation was similar between the two groups (p = 0.46) whereas mean time of fluoroscopy was significantly longer in IM fixation compared to plating (p < 0.001). There was a slight but significant difference in ROM degrees between the two groups (p = 0.005). Mean quick DASH score was significantly lower in IM fixation than that in plating (p< 0.001) whereas there was no significant difference in constant shoulder scores between the two groups (p = 0.06). Time to bony union was also shorter in IM fixation compared to plating (p< 0.001).
30	Lee 2007[48]	prospective RCT	62	59	Anterosuperior plating with DCP, tubular and reconstruction plates 30 Follow up duration: 30 months	Knowles pins 32 Follow up duration: 30 months	The mean shoulder score of the Knowles pinning was 85 points and the plating was 84 points (P = .7). Knowles pinning requires significantly shorter operative time (P < .001), smaller wound size (P < .001), shorter hospital stay (P = .03), less meperidine use (P = .02), lower complication rate (P = 0.04), and less symptomatic hardware (P = .015).
31	Simek 2020[6]	prospective RCT	60	Age not specified	Plate 30 patients Follow up duration: 12 months	ESIN with Titanium elastic nail fixation: 30 patients Follow up duration: 12 months	The time to clavicle fracture was comparable in both arms (approximately 3 months). Functional Constant score was comparable in both arms (p = 0.268) shorter incision (p<0.001), longer radiation exposure (p<0.001) and higher radiation dose (p<0.001) in ESIN group, compared with plate fixation group.

### Surgical versus non-operative management

Of the 3094 patients, the mean age is 36.7 years old with most displaced clavicle fractures of the midshaft, AO/OTA classification 2B1 and 2B2. Table 2 shows the summary of the meta-analysis results. Compared with non-operative treatment, surgical treatment is associated with better clinical recovery in terms of lower overall DASH score (standard-mean difference -0.22 (95% CI -0.36 to -0.07; p = 0.003)) [11, 23, 24, 27, 29, 31, 32, 35], higher overall CMS (standard-mean difference 0.44 (95% CI 0.17–0.72; p = 0.001) [11, 21, 23, 24, 26, 27, 29, 31–33, 35], shorter time to bone union (non-adjusted standard-mean difference -2.83, 95% CI -4.59 to -1.07, p = 0.002; adjusted standard-mean difference -0.69, 95% CI -0.97 to -0.41, p<0.001) [21, 32, 33, 35], and relatively low chance of bone-related major complications including non-union, malunion and implant failure (risk ratio is 0.21 (95% CI 0.1 to 0.42; p<0.001) [7, 11, 21, 23, 26, 28–33, 35, 49].

**Table 2:** **a.** Summary of the meta-analysis result. **b.** Comparison of the standard-mean difference (SMD) and mean difference (MD) of DASH Score and Constant-Murley Score at different time points between surgical treatment and conservative treatment.

**a.** Summary of the meta-analysis result				
	**DASH (SMD)**	**CmS (SMD)**	**Time to union (SMD)**	**Bone-related major complication (odd ratio)**
**Surgical vs non-surgical**	-0.22 (95% CI -0.36 to -0.07; p = <0.01)	0.44 (95% CI 0.17–0.72; p = <0.01)	-2.83 (95% CI -4.59 to -1.07; p = 0.002)	0.16 (95% CI 0.07–0.35; p<0.01)
**Intra-surgical: Plate vs Nail**	0.01 (95% CI -0.31 to 0.33, p = 0.94)	0.09 (95% CI -0.27 to 0.45; p = 0.61)	0.82 (95% CI -0.08 to 1.71; p = 0.07)	0.97 (95% CI 0.37 to 2.56; p = 0.96)
**b. Comparison of the standard-mean difference (SMD) and mean difference (MD) of DASH Score and Constant-Murley Score at different time points between surgical treatment and conservative treatment.**				
	**Early (3 months)**	**Intermediate (6–12 months)**	**Late (24 months)**	**Overall**
**i) Standard-mean difference**				
**DASH**	-0.12 (95% CI -0.34 to 0.09; p = 0.25)	-0.16 (95% CI -0.30 to -0.02; p = 0.02)	-0.51 (95% CI -0.73 to -0.28; p<0.01)	-0.22 (95% CI -0.36 to -0.07; p<0.01)
**CMS**	-0.12 (95% CI -0.13 to 0.37; p = 0.35)	0.18 (95% CI -0.09 to 0.44; p = 0.19)	1.03 (95% CI 0.39 to 1.68; p<0.01)	0.44 (95% CI 0.17–0.72; p<0.01)
**ii) Mean difference**				
**DASH**	-1.22 (95% CI -3.91 to 1.47; p = 0.37)	-0.97 (95% CI -2.42 to 0.48; p = 0.19)	-4.24 (95% CI -7.03 to -1.45; p<0.01)	-1.72 (95% CI -2.93 to -0.51; p<0.01)
**CMS**	1.12 (95% CI -1.65 to 3.89; p = 0.43)	2.84 (95% CI -1.18 to 6.86; p = 0.17)	5.77 (95% CI 1.63 to 9.91; p<0.01)	3.64 (95% CI 1.09 to 6.19; p<0.01)

CMS: Constant-Murley Score.

DASH: Disability Arm Shoulder Hand Score.

SMD: Standard-Mean Difference.

**Fig 2:**
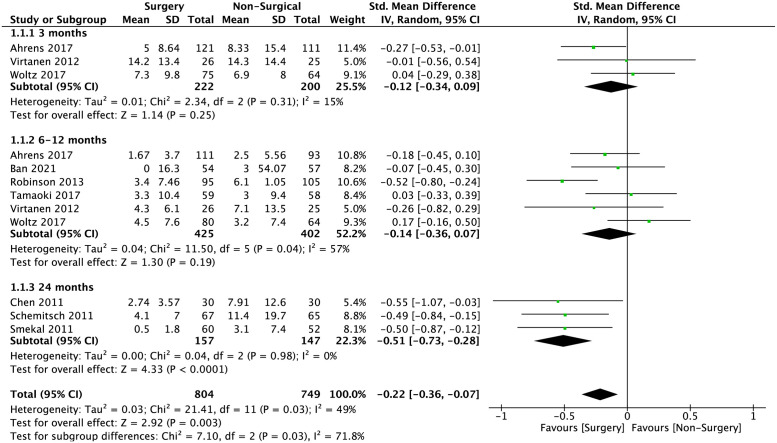
Meta-analysis of overall DASH score at ≤3 months, 6–12 months and ≥24 months between surgical interventions and non-surgical interventions in the pooled studies. Standard-mean difference is -0.22 (95% CI -0.36 to -0.07; p = 0.003).

The functional outcomes comparison between surgical and non-surgical management at different time points is shown in Fig 2. The early outcome (≤3 months after treatment) standard-mean difference of DASH score was -0.12 (95% CI -0.34 to 0.09; p = 0.25), intermediate outcome (6–12 months after treatment) and late outcome (≥24 months after treatment) difference was -0.16 (95% CI -0.30 to -0.02; p = 0.02) and -0.51 (95% CI -0.73 to -0.28; p<0.001) respectively. The overall standard-mean difference of DASH score was -0.22 (95% CI -0.36 to -0.07; p = 0.003).

**Fig 3:**
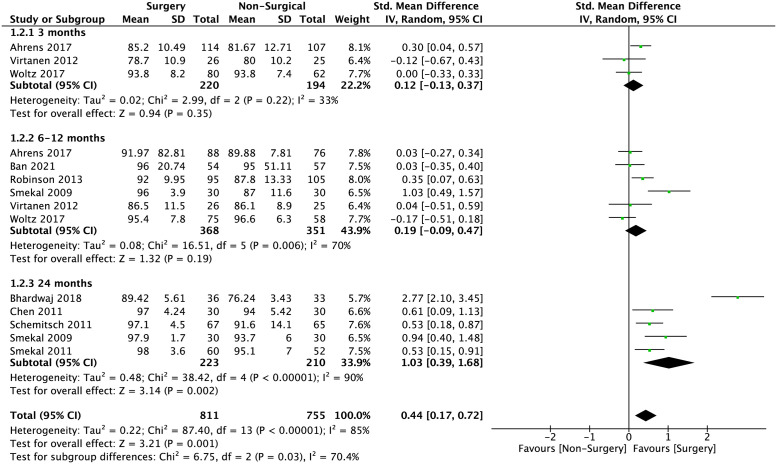
Meta-analysis of overall CMS at ≤3 months, 6–12 months and ≥24 months between surgical interventions and non-surgical interventions in the pooled studies. Standard-mean difference is 0.44 (95% CI 0.17–0.72; p = 0.001).

The standard-mean difference of CMS at early, intermediate, and late outcomes were -0.12 (95% CI -0.13 to 0.37; p = 0.35), 0.18 (95% CI -0.09 to 0.44; p = 0.19) and 1.03 (95% CI 0.39 to 1.68; p = 0.002), respectively. The overall standard-mean difference in CMS is 0.44 (95% CI 0.17–0.72; p = 0.001) as shown in Fig 3.

**Fig 4:**
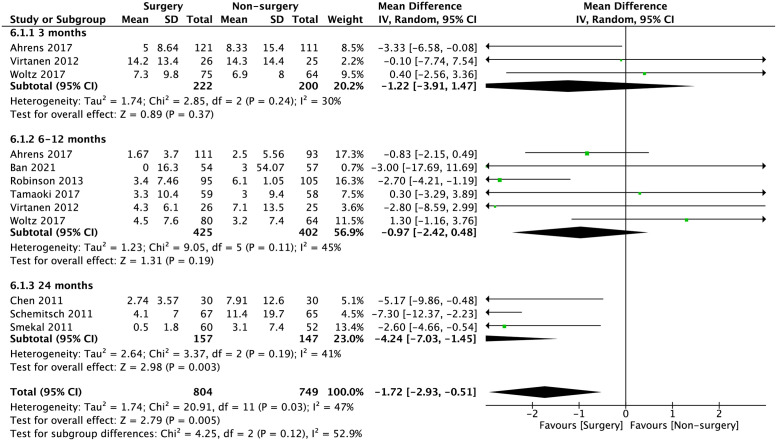
Meta-analysis of mean difference of DASH score between surgical intervention and non-surgical intervention in the pooled studies. Overall -1.72 (95% CI -2.93 to -0.51; p = 0.005); early (3-months) -1.22 (95% CI -3.91 to 1.47; p = 0.37); intermediate (6–12 months) -0.97 (95% CI -2.42 to 0.48; p = 0.19); late (24 months) -4.24 (95% CI -7.03 to -1.45; p = 0.003).

**Fig 5:**
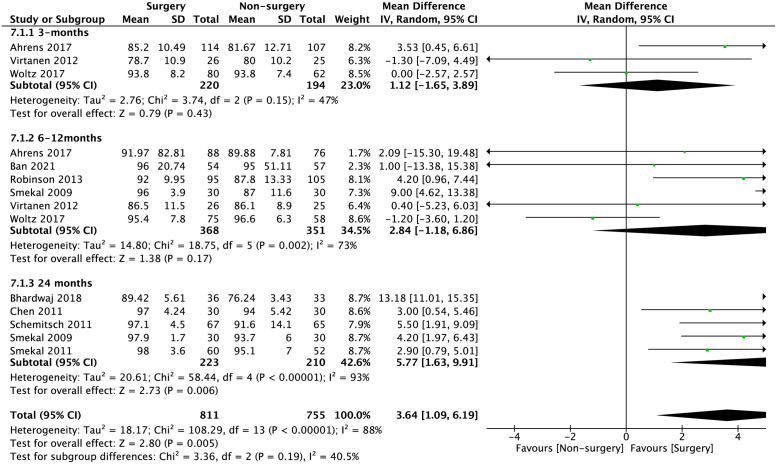
Meta-analysis of mean difference of CMS between surgical intervention and non-surgical intervention in the pooled studies. Overall, 3.64 (95% CI 1.09 to 6.19; p = 0.005) Early (≤3-months) 1.12 (95% CI -1.65 to 3.89; p = 0.43); Intermediate (6–12 months) 2.84 (95% CI -1.18 to 6.86; p = 0.17); Late (≥24 months) 5.77 (95% CI 1.63 to 9.91; p = 0.006).

The mean differences of DASH score and CMS between surgical and non-surgical management is shown in Figs 4 and 5. The mean difference of overall, early, intermediate, and late DASH scores are: -1.72 (95% CI -2.93 to -0.51; p = 0.005), -1.22 (95% CI -3.91 to 1.47; p = 0.37), -0.97 (95% CI -2.42 to 0.48; p = 0.19 and -4.24 (95% CI -7.03 to -1.45; p = 0.003), respectively. The mean difference of overall, early, intermediate, and late CMS are 3.64 (95% CI 1.09 to 6.19; p = 0.005); 1.12 (95% CI -1.65 to 3.89; p = 0.43); 2.84 (95% CI -1.18 to 6.86; p = 0.17) and 5.77 (95% CI 1.63 to 9.91; p = 0.006).

**Fig 6:**
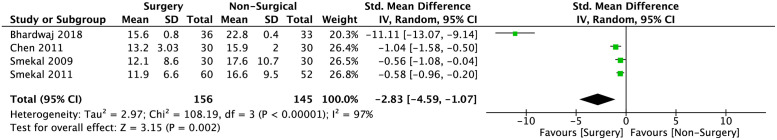
Meta-analysis of time to bony union between surgical and non-surgical intervention in pooled studies. Standard-mean difference is -2.83 (95% CI -4.59 to -1.07; p = 0.002).

**Fig 7:**
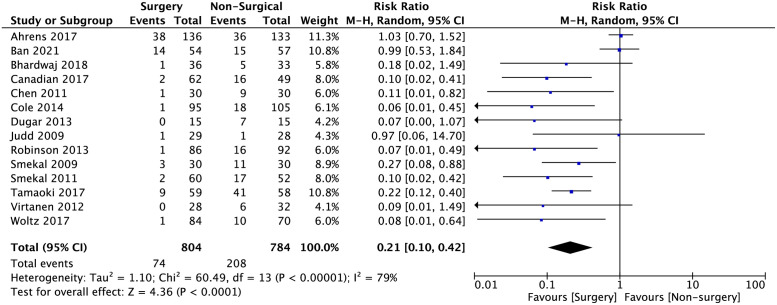
Meta-analysis of risk ratio of bone-related major complications (including bone non-union, malunion, implant failure) between surgical and non-surgical intervention in pooled studies. The risk ratio is 0.21 (95% CI 0.10 to 0.42; p<0.001).

Pooled data analysis for secondary outcomes in time to bony union and risk ratio for bone related complications are shown in Figs 6 and 7. A statistically significant reduction in time to achieve bony union was seen in the surgical intervention group with a standard-mean difference of -2.83 (95% CI -4.59 to -1.07; p = 0.002). The risk of bone-related major complications, including bone non-union, malunion and implant failure was lower in the surgical compared to the non-surgical group with a risk ratio of 0.21 (95% CI 0.1 to 0.42; p<0.001).

### Surgical plate fixation versus intramedullary nailing

Subgroup analysis within the surgical intervention group on the type of fixation and approach used was also done (See S1 File). Surgical outcomes by fixation of plate compared to intramedullary nailing were compared. Functional outcomes as seen in **S1** and **S2 of** S1 File, showed no difference in DASH score, standard-mean difference is 0.01 (95% CI -0.31 to 0.33; p = 0.94) [37, 38, 40, 46, 47], and CMS score, standard-mean difference -0.09 (95% CI -0.27 to 0.45; p = 0.61) [36–38, 41, 42, 47]. Secondary outcomes with time to bony union standard-mean difference 0.82, (95% CI -0.08 to 1.71, p = 0.07) [38, 41, 42, 47] and bone-related complications (risk ratio 0.98, 95% CI 0.39 to 2.45; p = 0.97) [36, 38–42, 46, 47] were also not significant (**S3** and **S4 of** S1 File, **respectively**).

### Minimally invasive surgery versus conventional plate fixation

Different methods of plating has been described in clinical trials including the comparison between minimally invasive surgery (MIS) and conventional plate fixation. Jiang and Qu showed that a lower complication rate with less scar dysesthesia, hypertrophic scarring and shoulder pain was observed in the MIS group [44]. However, the average time to achieve bone union was similar in both groups (13 weeks vs. 12 weeks in the MIS group compared with conventional plating, p>0.05). These results were consistent with the findings of Kim et al., who showed that MIS was not associated with a better clinical and functional outcome in terms of time to bone union and Constant-Murley score [43]. The advantages of MIS were shorter operative time (52.33 ± 13.87 vs 110.33 ± 25.39 minutes, p<0.001) and scar length (64.95 ± 3.19 vs 99.39 ± 15.98mm, p<0.001).

## Discussion

This systematic review and meta-analysis aimed to evaluate the difference in treatment choice for midshaft clavicular fractures with regards to early, intermediate, and late outcomes. Previous meta-analysis of 15 randomised controlled trials in 2015 showed that surgical and non-surgical management had similar functional outcomes and complication rates after 1 year follow-up [13]. Another meta-analysis in 2019, also with 1-year follow-up still showed no difference in functional outcomes, but low rate of revision surgery and complications with surgical intervention [14]. Another recent meta-analysis with 14 RCT including 1546 patients showed an improvement of functional scores (DASH and CMS) and lower complication rates [15]. However, this meta-analysis defined short-term as 6 weeks with lacking data for CMS functional scores [15]. Moreover, time to bone union may range from 6 weeks to 13 weeks and short-term outcomes before bony union may be inaccurately assessed as functional rehabilitation and return may not yet be attainable [7, 11, 50]. Long-term data was broadly grouped and defined as >9 months in this study. To date, no meta-analysis has investigated into the difference between surgical and non-surgical interventions specifically for more than 2 years. This is the first meta-analysis to compare the difference in clinical outcomes of surgical interventions and non-surgical treatment with stratification into early (≤3 months), intermediate (6–12 months) and late (≥24 months) time-points to provide a holistic picture for treatment outcomes in the long term.

This meta-analysis of 31 RCT involving 3094 patients showed that surgical fixation is a better treatment choice for midshaft clavicular fractures, compared with non-operative treatment. Better outcomes in terms of standard-mean difference of DASH score, CMS, time to union and risk ratio for associated complications were observed in surgical fixation group. Surgical intervention provides better mechanical stability, and we postulate a more rapid recovery of shoulder function-related scores. The overall and long term (≥24 months) DASH scores in operative group were significantly better than the non-operative group; with a long term improvement by 4.24 points and a standard-mean difference suggestive of a large effect size. This is similarly true in the overall CMS and late CMS outcomes with a significant improvement by a difference of 5.77 score after 2 years. Surgical intervention also favours earlier restoration of shoulder function scores (both DASH and CMS) in the early and intermediate outcomes, but without statistical significance, contrary to what we postulated. Surgical intervention is associated with a shorter time to bone union (SMD -0.69, 95% CI -0.97 to -0.41, p<0.001) and lower risk of bone-related complications (risk ratio 0.21, 95% CI 0.1–0.42; p<0.001).

However, the difference and improvement in the DASH and CMS functional scores albeit significant did not reach the minimal clinically significant difference (MCID). Axelrod et al. and Franchignoni et al. showed that the established threshold for MCID of DASH score is between 8 and 10 points [19, 51]; and the MCID of Constant score to be at a score 10.4 [18]. However, the mean difference in DASH and CMS between intermediate and late time-points continued to improve with the difference in mean CMS score doubling; suggesting that the clinical benefits of surgery may be more obvious in the long-term. The discussion of potential long-term functional benefits with patients in day-to-day clinical practice can help them decide on their choice of treatment.

Given that more evidence suggests surgical intervention has better outcomes than non-operative treatment—ways to optimize surgical approach for midshaft clavicular fractures needs to be investigated. In this study we also compared the surgical outcomes between intramedullary nail (IM nail) and plate fixation in terms of functional DASH scores and CMS, time to union and fracture related complications rate. However, no statistically significant difference was found. Both implants are safe options for surgical fixation but subject to variability in fracture pattern and surgeon preference [38, 42]. IM nail has the theoretical advantage of preserving the periosteal blood supply, but carries the higher risk of pin migration and non-union with less rigid fixation [36]. Fuglesang et al. recommended the use of plate fixation in patients with comminuted fractures due to a quicker recovery and IM nail for non-comminuted fractures since it is associated with shorter operative time, lower infection and implant failure rate, better cosmetic outcomes and earlier return to work [40, 41]. IM nailing is inherently difficult to be performed in comminuted fractures and may not be appropriate for such fracture patterns. In addition, Hulsman et al. found that implant removal rate in IM nailing was higher (82% vs 50%) compared to plate fixation, with a relative risk of 1.65 (95% CI 1.24–2.19; p<0.001) [46].

There were several limitations to this study. Despite all included studies being RCTs, there was significant heterogeneity between studies including patient population and surgical techniques. For example, different type of implants used including: anatomical and non-anatomical locking plates, dynamic compression plates and IM nails. Also differences in surgical approaches with superior or anterior plating. Further network meta-analyses are required to investigate the optimum surgical fixation method and approach on functional outcomes. Other commonly seen complications such as infections (superficial and deep), poor shoulder cosmesis and rate of implant removal for symptomatic hardware were not assessed in this study. Lastly, the sensitivity of DASH and CMS functional scores used in this study in detecting subtle clinically significant differences has also been raised into question. Differences in healthy young adults may not be detected for example, small power deficits with a shortened muscular lever arm with the loss of clavicle length in non-operative management [52, 53].

## Conclusion

Surgical intervention for midshaft clavicle fractures is associated with better clinical outcomes compared with non-surgical management for midshaft clavicular fractures in terms of functional improvement in DASH and CMS, with benefit seen in long-term results albeit not to the MCID. Time to bone union and fracture related complications were also improved with surgical intervention. There was no significant difference in outcomes between surgical fixation with plating or intramedullary nailing. Further studies are needed to determine the optimal surgical fixation method and approach.

## Supporting information

S1 Checklist PRISMA 2009 checklist. (DOC)

S1 File File contains all the supporting files and figures. (DOCX)
