# Supplementary material for: Operative management of midshaft clavicle fractures demonstrates better long-term outcomes: A systematic review and meta-analysis of randomised controlled trials
Source: PLoS One. 2022 Apr 29;17(4):e0267861. doi: 10.1371/journal.pone.0267861 (PMC9053819; doi:10.1371/journal.pone.0267861)
Supplement: S1 File — (DOCX) [file pone.0267861.s002.docx]

**Operative Management of Midshaft Clavicle Fractures Demonstrates Better Long-Term Outcomes: A Systematic Review and Meta-analysis of Randomised Controlled Trials**

Michael Zhipeng Yan ^1^; Wing-sze Yuen^1^; Sung-ching Yeung^1^; Christie Wong Wing-yin^1^; Sonia Choi-ying Wong^1^;Walter Wang Si-qi^1^; Elaine Tian^1^; Shireen Rashed^1^, ; Colin Shing Yat Yung^1^, Christian Xinshuo Fang^1*^

^1^ Department of Orthopaedics and Traumatology, The University of Hong Kong, Queen Mary Hospital, Hong Kong Special Administrative Region, China

^*^Corresponding authors

Supplementary Appendix

**
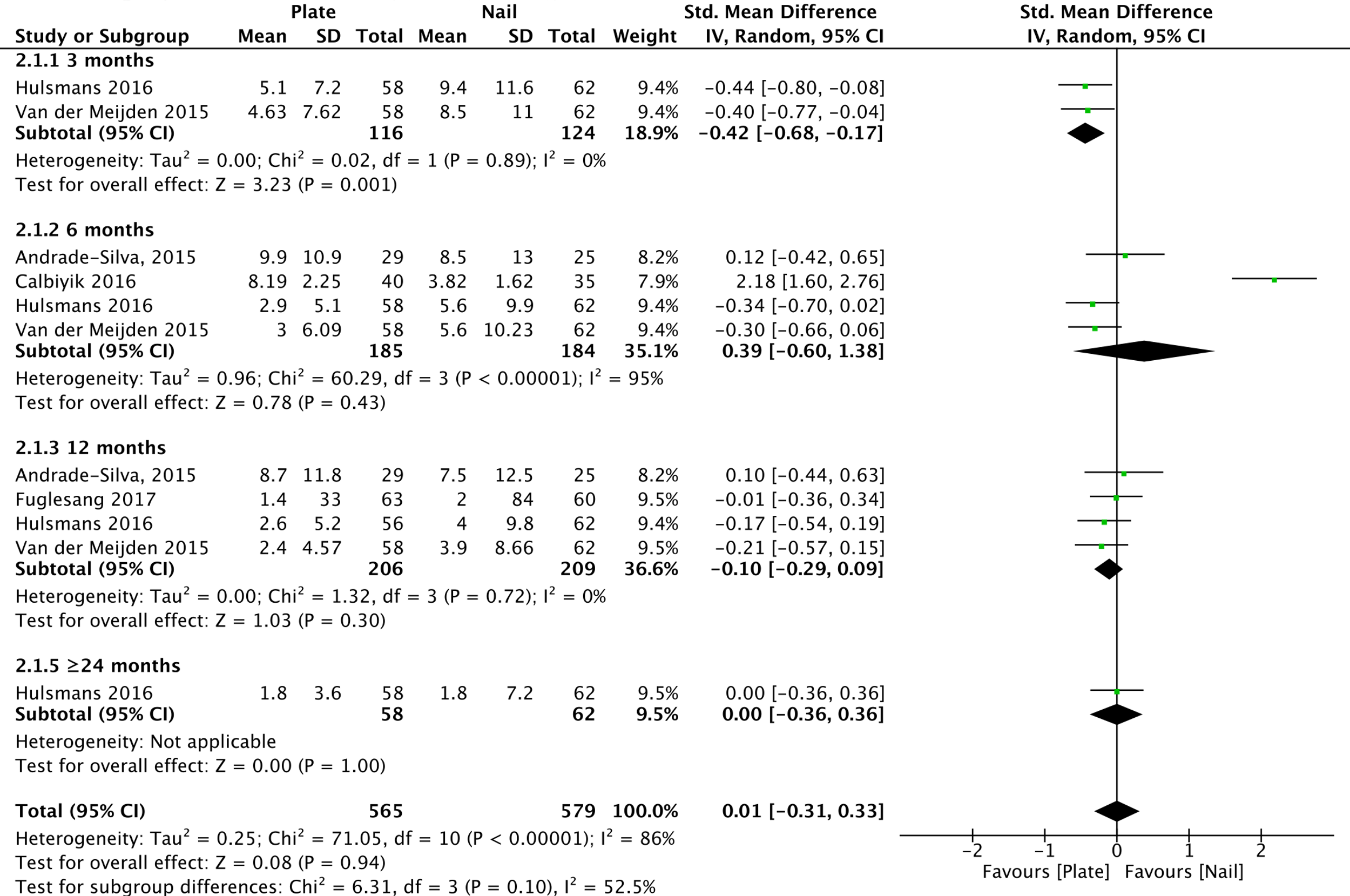
S1.** Meta-analysis of DASH Score outcomes comparing between plate fixation versus intramedullary nailing at 3, 6, 12, and ≥24 months in pooled studies. The standard-mean difference was 0.01 (95% CI -0.31 to 0.33; p=0.94).

**
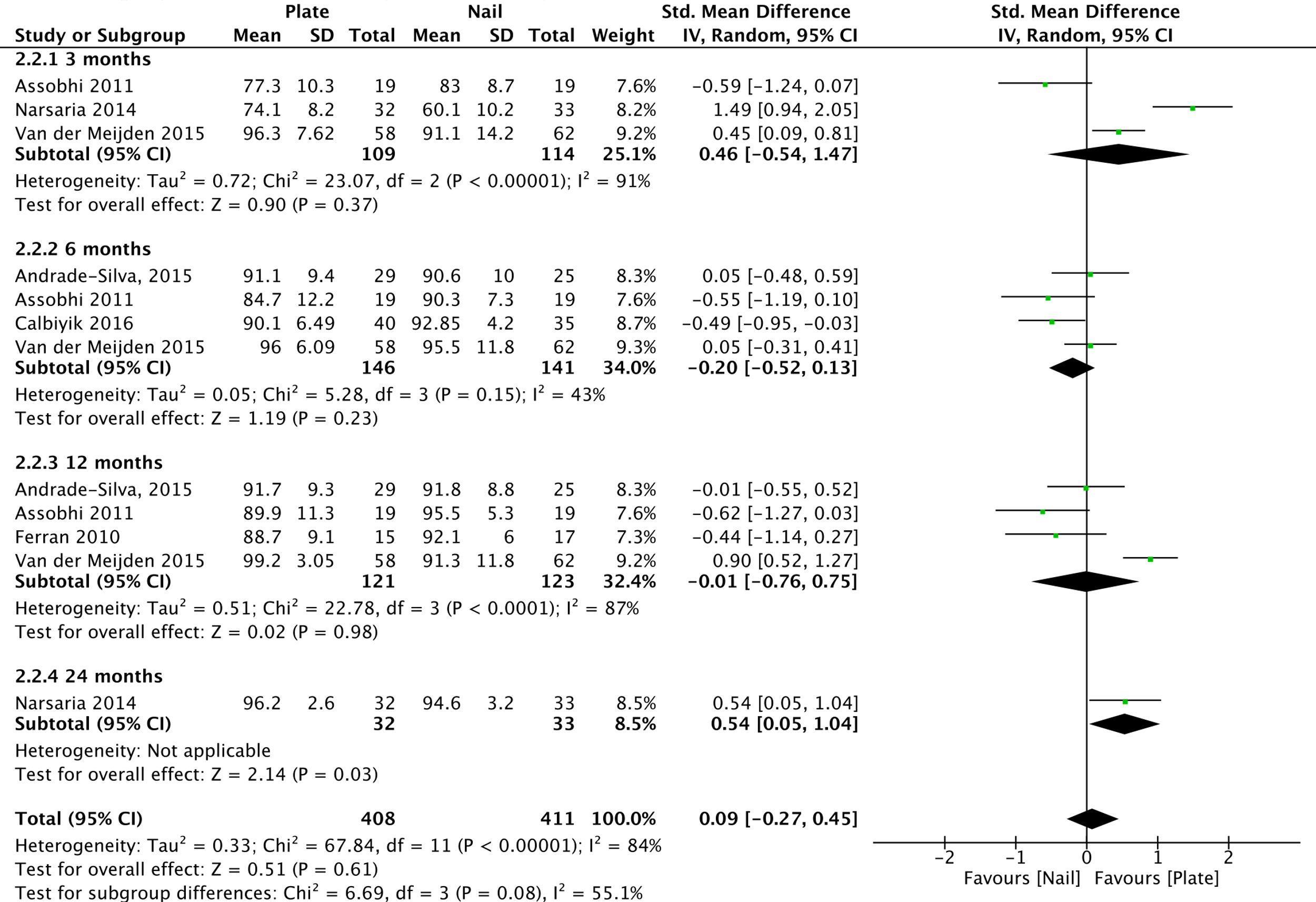
S2.** Meta-analysis of CMS outcomes comparing between plate fixation and intramedullary nailing in pooled studies at 3, 6, 12 and ≥24 months. The standard mean difference was 0.09 (95% CI -0.27 to 0.45; p=0.61).

**
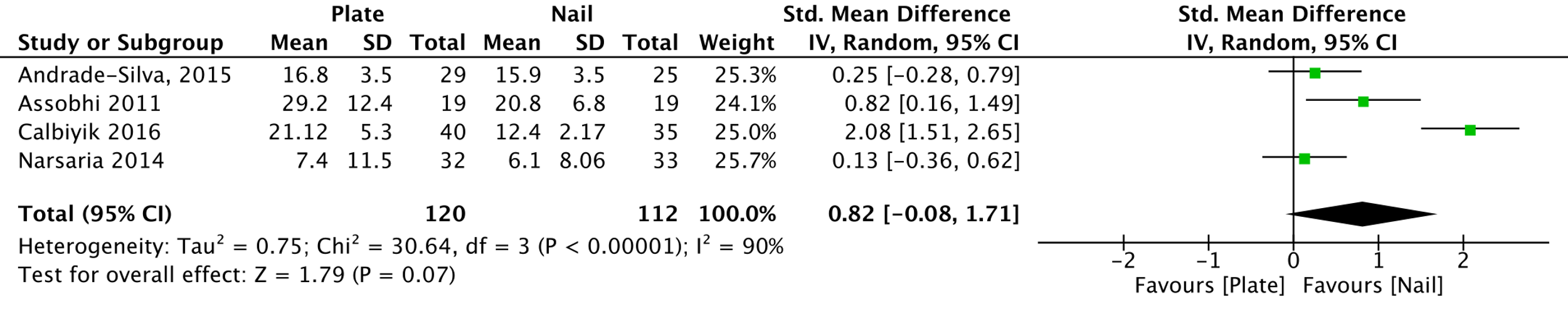
S3.** Meta-analysis of time to bony union comparison between plate fixation and intramedullary nailing in pooled studies. The standard mean difference is 0.82 (95% CI -0.08 to 1.71; p=0.07).

**
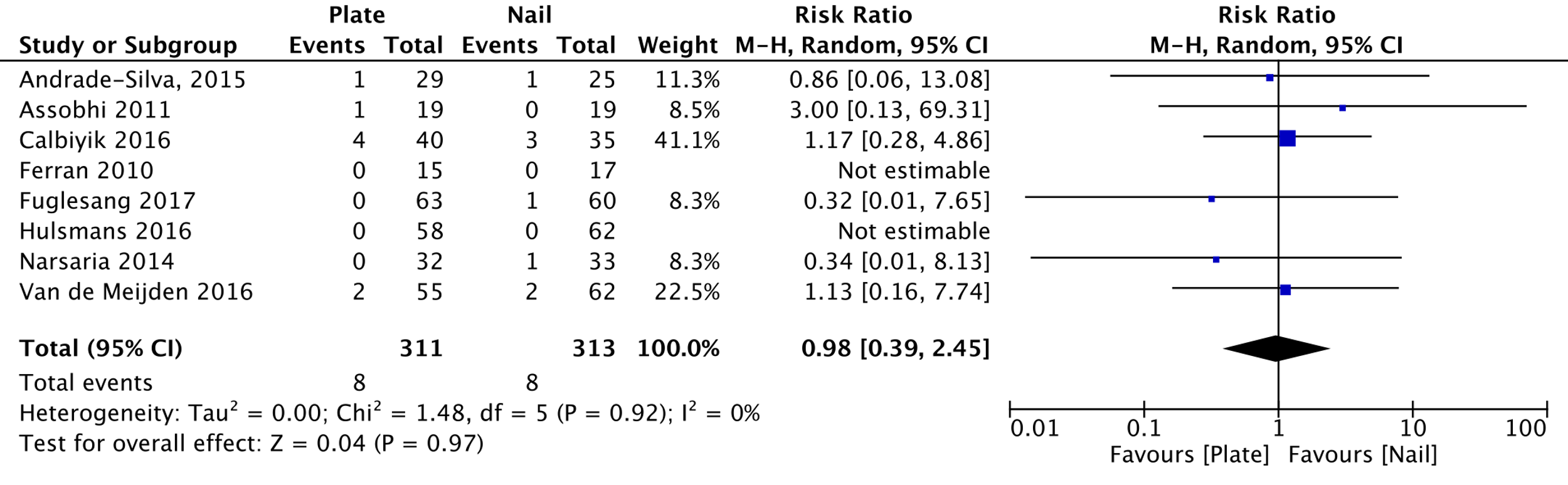
S4.** Meta-analysis of bone-related major complications (including bone non-union, malunion and implant failure) between plate fixation and intramedullary nailing in pooled studies. The risk ratio for complications is 0.98 (95% CI 0.39 to 2.45; p=0.97).

**S5. Distribution of implants in the pooled study comparing between surgical outcomes beween conservative and operative treatment.**

**LCP: Locking Clavicle Plate Fixation (in blue);**

**DCP: Dynamic Compression Plate Fixation (in yellow) ;**

**(LCP+DCP): Hybrid Plate Fixation in green);**

**Nail Fixation (in orange);**

**Pin Fixation (in purple);**

**Screw Fixation (in red).**

**S6. Distribution of Implants in the pooled study of intra-surgical group comparison between Nail and Plate.**

IM nail: Intramedullary Nail Fixation (in blue); ESIN + Titanium Elastic Nail Fixation (in yellow) ; Knowles Pin Fixation (in green)


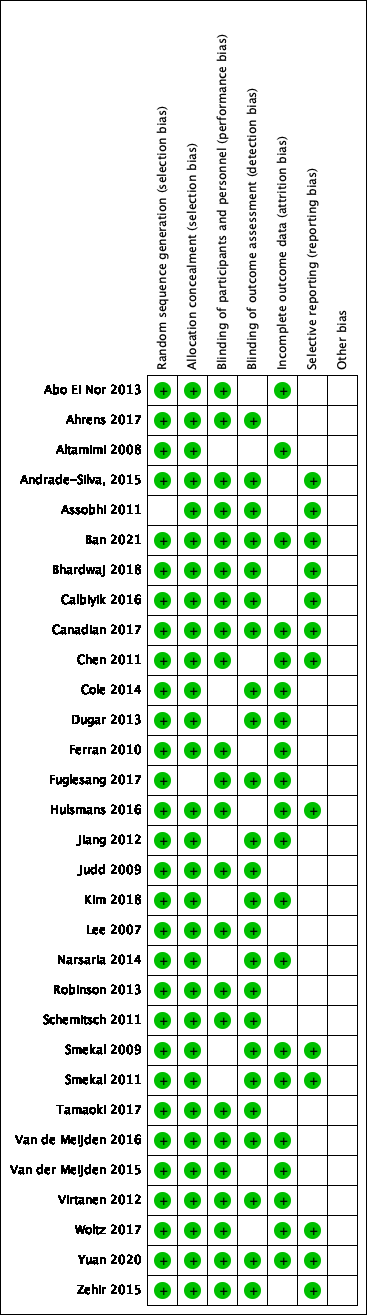


S7. Risk of Bias summary


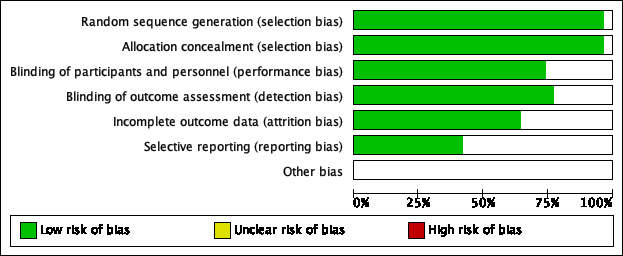


S8. Risk of bias graph


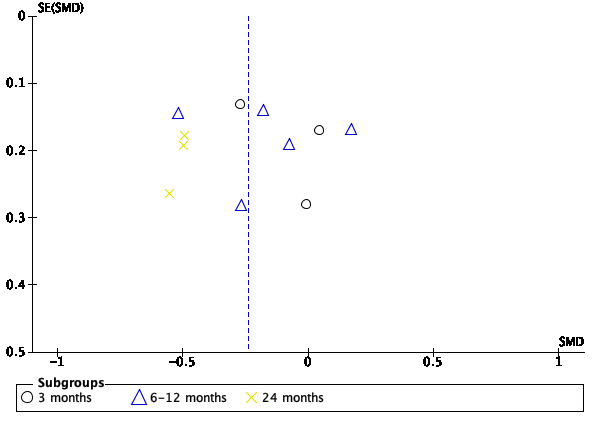


S9a. Funnel plot of operative treatment versus non-operative treatment treatment.(Overall DASH score)


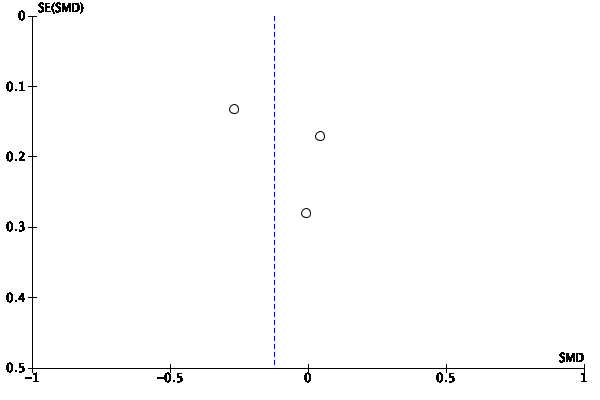


S9b. Funnel plot of operative treatment versus non-operative treatment treatment.(Early DASH score at 3 months)


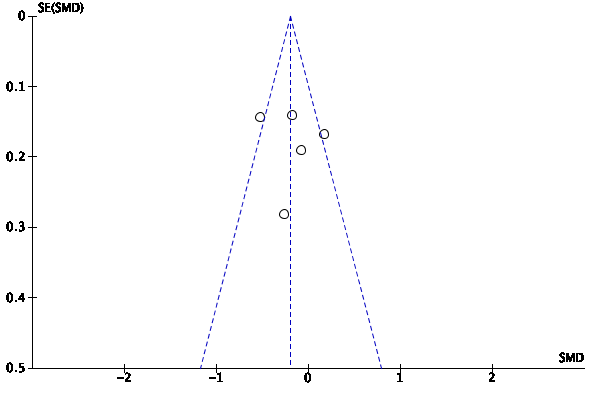


S9c. Funnel plot of operative treatment versus non-operative treatment.(Intermediate DASH score at 6-12 months)


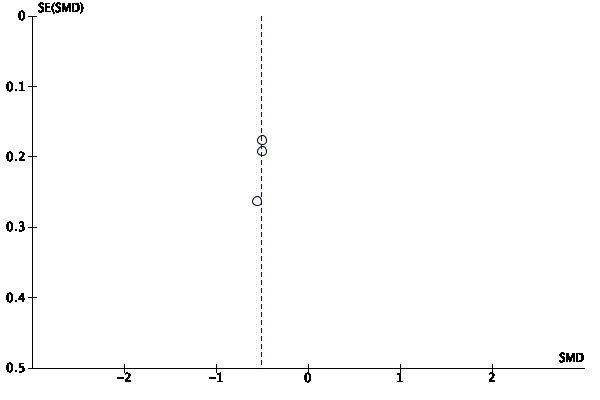


S9d. Funnel plot of operative treatment versus non-operative treatment.(Late DASH score at 24 months)


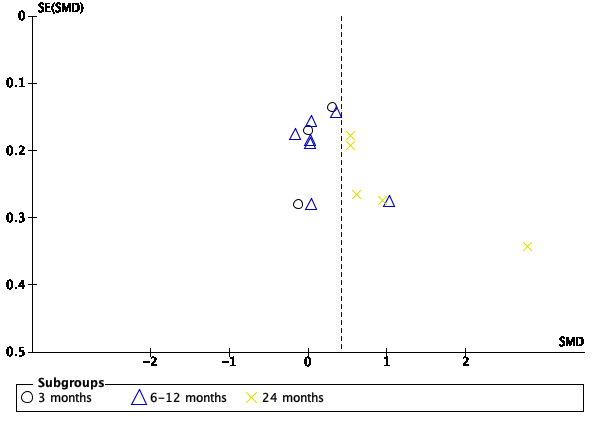


S10a. Funnel plot of operative treatment versus non-operative treatment treatment.(Overall CMS)


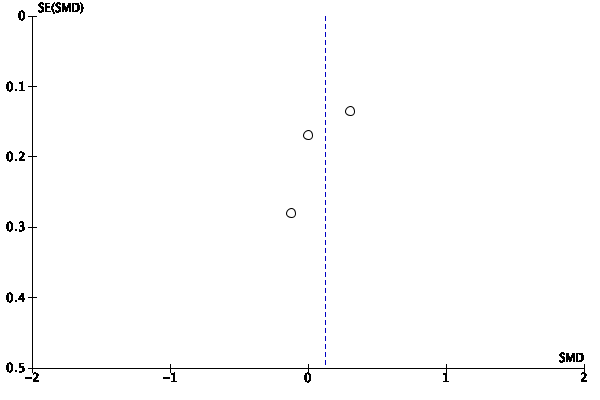


S10b. Funnel plot of operative treatment versus non-operative treatment.(early CMS at 3 months)


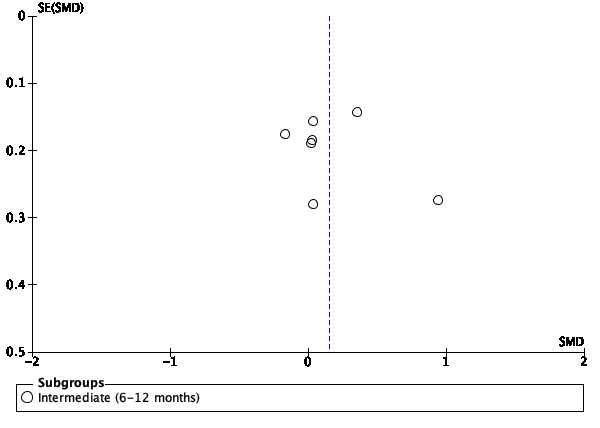


S10c. Funnel plot of operative treatment versus non-operative treatment.(Intermediate CMS at 6-12 months)


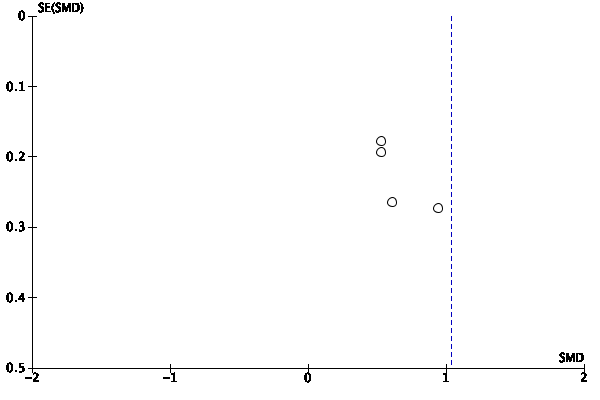


S10d. Funnel plot of operative treatment versus non-operative treatment.(Late CMS at 24 months)


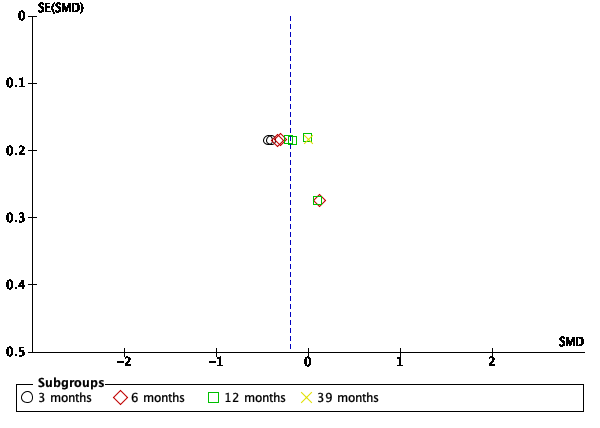


S11. Funnel plot of intra-surgical comparison: surgical plate fixation versus intramedullary nail fixation.(Overall DASH score)


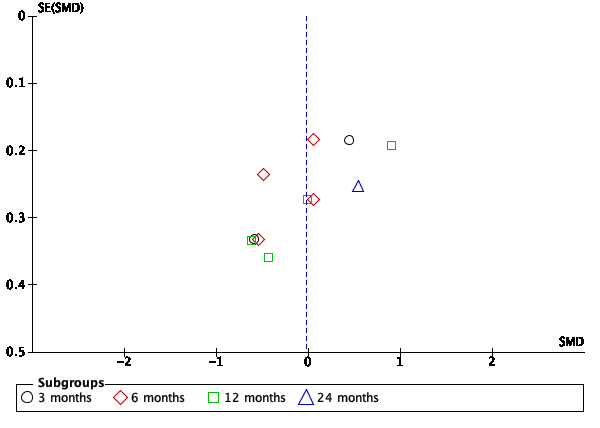


S12. Funnel plot of intra-surgical comparison: surgical plate fixation versus intramedullary nail fixation.(Overall CMS)


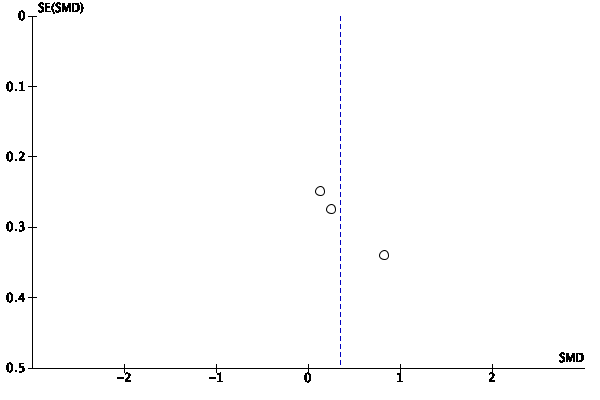


S13. Funnel plot of intra-surgical comparison: surgical plate fixation versus intramedullary nail fixation.(Time to union)


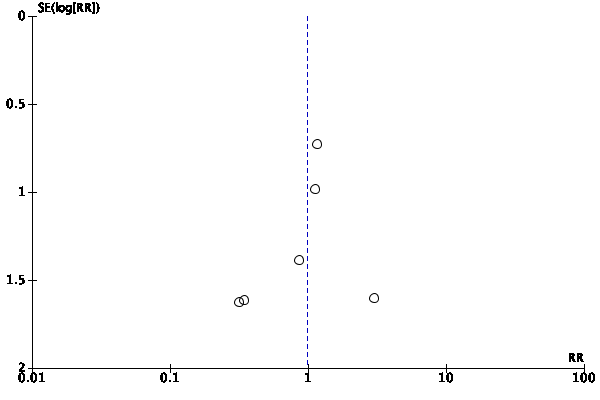


S14. Funnel plot of intra-surgical comparison: surgical plate fixation versus intramedullary nail fixation.(Risk ratio of bone-related complications rate)
